# The pretreatment albumin to globulin ratio predicts chemotherapeutic outcomes in patients with unresectable metastatic colorectal cancer

**DOI:** 10.1186/s12885-015-1375-x

**Published:** 2015-05-02

**Authors:** Masatsune Shibutani, Kiyoshi Maeda, Hisashi Nagahara, Hiroshi Ohtani, Yasuhito Iseki, Tetsuro Ikeya, Kenji Sugano, Kosei Hirakawa

**Affiliations:** Department of Surgical Oncology, Osaka City University Graduate School of Medicine, 1-4–3 Asahi-machi Abeno–ku, Osaka, Japan

**Keywords:** Colorectal cancer, Prognosis, Unresectable, Chemotherapy, Albumin to globulin ratio

## Abstract

**Background:**

The pretreatment albumin to globulin ratio (AGR) has been reported to correlate with the long-term survival in patients with various cancers. However, there are no reports regarding the correlation between the pretreatment AGR and chemotherapeutic outcomes in patients with unresectable metastatic colorectal cancer. The aim of this study was to evaluate the prognostic significance of the pretreatment AGR in patients with unresectable metastatic colorectal cancer.

**Methods:**

A total of 66 patients with unresectable metastatic colorectal cancer who underwent palliative chemotherapy for metastatic tumors were enrolled. The AGR was calculated as follows: Albumin/(Total protein - Albumin).

**Results:**

The median pretreatment AGR was 1.254 (range: 0.849-1.840). We set 1.25 as the cut-off value based on the receiver operating characteristic curve. Based on the cut-off value of 1.25, 34 patients were classified into the high-AGR group and 32 patients were classified into the low-AGR group. The high-AGR group had a significantly higher chemotherapeutic disease control rate (p = 0.040) and better progression-free survival (p = 0.0171) and overall survival (p = 0.0360) rates than the low-AGR group. According to a multivariate analysis of survival, the AGR was identified to be an independent prognostic factor for progression-free survival (Hazard Ratio: 2.662, 95% Confidence Interval: 1.085-6.631, p = 0.033) and overall survival (Hazard Ratio: 2.247, 95% Confidence Interval: 1.069-4.722, p = 0.033).

**Conclusions:**

The pretreatment AGR is a useful prognostic marker in patients with unresectable metastatic colorectal cancer who receive palliative chemotherapy.

## Background

Colorectal cancer is one of the most common causes of cancer-related death worldwide [1]. In particular, patients with unresectable metastatic colorectal cancer have a worse prognosis. Although there have been major advances in the treatment of unresectable metastatic colorectal cancer within the last 10 years, including the introduction of new cytotoxic and molecular targeted therapies [2-5], the response to palliative chemotherapy varies and many patients die in the early stage after the initiation of treatment due to the ineffectiveness of chemotherapy. Therefore, it is necessary to detect biomarkers predicting the chemotherapeutic response and survival outcomes.

Markers of the systemic inflammatory response, such as the neutrophil to lymphocyte ratio (NLR), C-reactive protein level and Glasgow prognostic score (GPS), have been investigated as prognostic factors in colorectal cancer [6-11]. Recently, the albumin to globulin ratio (AGR), which also reflects the degree of systemic inflammation, has been reported to be a prognostic marker in patients with colorectal [12], lung [13] and breast [14] cancers.

Albumin and globulin are the two major components of serum proteins. A decreased albumin level and increased globulin level have been reported to reflect chronic inflammation [14-16]. Because systemic inflammation has been shown to cause an increase in the levels of various proinflammatory cytokines, which subsequently promote progression of the tumor due to changes in the cancer microenvironment [17,18], a decreased AGR is thought to correlate with tumor progression.

A few previous studies have reported a correlation between the pretreatment AGR and long-term mortality. However, there are no reports on the relationship between the AGR and the chemotherapeutic outcome in patients with colorectal cancer.

The aim of this retrospective study was to evaluate whether the pretreatment AGR can be used as a predictor of chemotherapeutic outcomes and long-term mortality in patients with unresectable metastatic colorectal cancer.

## Methods

### Patients

We retrospectively reviewed a database of 66 patients who underwent palliative combination chemotherapy for unresectable colorectal cancer at the Department of Surgical Oncology of Osaka City University between 2006 and 2011. None of the patients had bowel obstruction, anemia or any other complications before chemotherapy.

The patient characteristics are listed in Table 1. The patient population consisted of 35 males and 31 females, with a median age of 63 years (range: 36 to 80). According to the definition of the Eastern Cooperative Oncology group performance status, 62 patients were classified as having a performance status of 0, three patients were classified as having a performance status of 1 and one patient was classified as having a performance status of 2. The median body mass index was 21.7 kg/m^2^ (range: 15.1-33.7). Thirty-six patients had primary tumors located in the colon and 30 had primary tumors located in the rectum. A total of 20 patients had metachronous unresectable cancer, and 46 patients had synchronous unresectable cancer. Forty-four patients had only one organ affected by metastasis and 22 patients had more than one organ affected by metastasis. All patients underwent combination chemotherapy with oxaliplatin or irinotecan plus 5-fluorouracil/leucovorin or a prodrug of 5-fluorouracil as first-line chemotherapy. In particular, 34 patients received 5-fluorouracil + leucovorin + oxaliplatin (FOLFOX), 19 patients received capecitabine + oxaliplatin (CapeOX), seven patients received 5-fluorouracil + leucovorin + irinotecan (FOLFIRI) and six patients received other regimens. Thirty-seven patients underwent chemotherapy combined with molecular targeted therapy.

**Table 1:** **Patient characteristics**

Age (years)	
Median (range)	63 (36–80)
Gender	
Male	35
Female	31
Performance status	
0/1/2	62/3/1
Body Mass Index (kg/m^2^)	
Median (range)	21.7 (15.1-33.7)
Location of primary tumor	
Colon	36
Rectum	30
Histological type	
Well, Moderately	58
Poorly, Mucinous	8
Detection of unresectable tumor	
Synchronous	46
Metachronous	20
The number of organs affected by metastasis	
One organ	44
More than one organ	22
Regimen of first-line chemotherapy	
FOLFOX	34
CapeOX	19
FOLFIRI	7
Others	6
Molecular targeted therapy	
No	29
Yes	37
AGR	
Median (range)	1.254 (0.849-1.840)
NLR	
Median (range)	2.407 (0.580-7.644)
GPS	
0/1/2	42/12/9

FOLFOX: 5-fluorouracil + leucovorin + oxaliplatin; CapeOX: capecitabine + oxaliplatin; FOLFIRI: 5-fluorouracil + leucovorin + irinotecan; AGR: albumin to globulin ratio; NLR: neutrophil to lymphocyte ratio; GPS: Glasgow prognostic score.

### Evaluation

Response evaluations were performed every eight weeks. Variation of approximately one week was regarded as allowable error. All patients were followed up with a physical examination, blood tests, including measurements of the levels of tumor markers, such as carcinoembryonic antigen (CEA) and carbohydrate antigen 19–9 (CA 19–9), computed tomography and ultrasonography. Some patients underwent positron emission tomography or colonoscopy as needed.

We adopted the response evaluation criteria in solid tumors to classify the treatment response as follows [19]: complete response, partial response, stable disease and progressive disease. The objective response was defined as complete response or partial response, while disease control was defined as complete response, partial response or stable disease. Progression-free survival was defined as the time from the date of initiation of first-line chemotherapy to disease progression. Overall survival was defined as the time from the date of initiation of first-line chemotherapy to death from any cause or the last contact.

Pretreatment blood samples were obtained within one week before the initiation of chemotherapy. The AGR was calculated as follows: Albumin/(Total protein - Albumin). The NLR was calculated from the blood samples by dividing the absolute neutrophil count by the absolute lymphocyte count. We defined the GPS according to previous reports, as follows [20]: the combination of an elevated C-reactive protein level (≥1 mg/dl) and hypoalbuminemia (<3.5 g/dl). Patients with both abnormalities were allocated a GPS of 2, while patients with only one of these abnormalities were allocated a GPS of 1 and patients with normal values for both parameters were allocated a GPS of 0.

### Statistical analysis

First, we used a receiver operating characteristic curve to determine the appropriate cut-off value. All patients were classified into two groups according to the AGR. The significance of correlations between the pretreatment AGR and the clinicopathological characteristics/chemotherapeutic response was analyzed using the χ^2^ test, Fisher’s exact test and Mann-Whitney’s U-test. The duration of survival was calculated according to the Kaplan-Meier method. Differences in the survival curves were assessed with the log-rank test. A univariate analysis was performed for each variable identified to be a potential predictor of mortality according to a Cox proportional hazards model. A multivariate analysis was also performed using a Cox proportional hazards model. All statistical analyses were conducted using the SPSS software package for Windows (SPSS Japan, Tokyo, Japan). Statistical significance was set at a value of *p* <0.05.

### Ethical consideration

This research was conformed to the provisions of the Declaration of Helsinki in 1995. All patients were informed of the investigational nature of this study and provided written informed consent. This retrospective study was approved by the ethics committee of Osaka City University.

## Results

### Classification according to the pretreatment inflammatory markers

**Figure 1:**
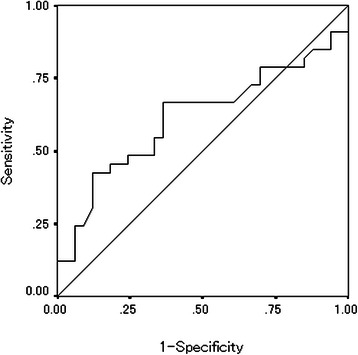
Receiver operating characteristic curve analysis of the AGR in the patients with unresectable metastatic colorectal cancer. Area under the curve =0.614, 95% Confidence interval = 0.474-0.754, p = 0.112.

We used the continuous variable AGR as the test variable and the 32-month survival (median survival time: 32 months) as the state variable. When we investigated the cut-off value for the AGR using the receiver operating characteristic curve, we found the appropriate cut-off value for the AGR to be 1.246 (sensitivity: 66.7% and specificity: 63.6%) (Figure 1). Therefore, we set 1.25 as the cut-off value and 34 patients were classified into the high-AGR group and 32 patients were classified into the low-AGR group.

We set 2.8 as the cut-off value for the NLR according to the previous report [6]. Based on a cut-off value of 2.8, 30 patients were classified into the high NLR group and 36 patients were classified into the low NLR group.

We set 2 as the cut-off value for GPS according to the previous report [21]. Based on a cut-off value of 2, 21 patients were classified into the high GPS group and 42 patients were classified into the low GPS group.

### Chemotherapeutic response

The distribution of the chemotherapeutic response after the first-line chemotherapy with reference to the AGR/NLR/GPS subgroup is shown in Table 2. The objective response rates did not differ according to the AGR (44.1% vs. 28.1%, p = 0.208). However, the high-AGR group had a significantly higher disease control rate than the low-AGR group (88.2% vs. 65.6%, p = 0.040). The NLR did not correlate with the chemotherapeutic response. The low GPS group had a significantly higher objective response rate than the high GPS group (42.7% vs. 12.5%, p = 0.034).

**Table 2:** **Treatment response to first-line chemotherapy according to the pretreatment AGR**

	AGR			NLR			GPS		
Response	High (n = 34)	Low (n = 32)	p-value	High (n = 30)	Low (n = 36)	p-value	Low (n = 54)	High (n = 9)	p-value
Complete response	2	0		0	2		2	0	
Partial response	13	9		9	13		20	1	
Stable disease	15	12		13	14		20	5	
Progressive disease	4	11		8	7		12	3	
Objective response rate	44.1%	28.1%	0.208	30.0%	41.7%	0.442	40.7%	12.5%	0.034
Disease control rate	88.2%	65.6%	0.040	73.3%	80.6%	0.562	77.8%	66.7%	0.434

AGR: albumin to globulin ratio.

### Survival analysis according to the pretreatment AGR

**Figure 2:**
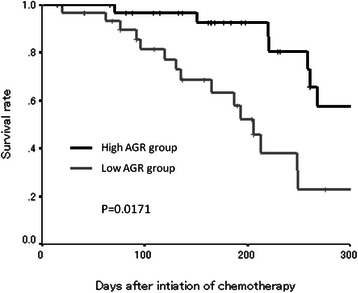
Kaplan-Meier survival curves for progression-free survival. The progression-free survival rate was significantly worse in the low-AGR group than in the high-AGR group (*p* = 0.0171).

**Figure 3:**
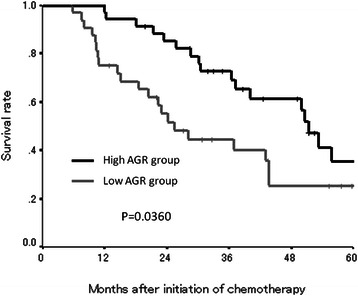
Kaplan-Meier survival curves for overall survival. The overall survival rate was also significantly worse in the low-AGR group (*p* = 0.0360).

The progression-free survival rate was significantly worse in the low-AGR group than in the high-AGR group (*p* = 0.0171) (Figure 2). Moreover, the overall survival rate was significantly worse in the low-AGR group (*p* = 0.0360) (Figure 3).

### Correlations between the pretreatment AGR and the clinicopathological factors

No relationships were observed between the pretreatment AGR and the clinicopathological factors except for the serum cholesterol concentration (p = 0.0011) (Table 3).

**Table 3:** **Correlations between the pretreatment AGR and the clinicopathological factors**

	AGR		
	Low	High	*p*-value
Performance Status			
0	31	31	
1/2	3	1	0.614
Body Mass Index (kg/m^2^)			
>18.5	30	28	
≤18.5	4	4	1.000
Location of primary tumor			
Colon	16	20	
Rectum	18	12	0.228
Histological type			
Well, Moderately	29	29	
Poorly, Mucinous	5	3	0.710
Detection of unresectable tumor			
Synchronous	22	24	
Metachronous	12	8	0.428
The number of organs affected by metastasis			
One organ	24	20	
More than one organ	10	12	0.603
Pretreatment CEA (ng/ml)			
>5	5	5	
≤5	29	27	1.000
Pretreatment CA19-9 (U/ml)			
>37	18	12	
≤37	15	19	0.222
Cholinesterase (IU/l)			
>235	2	7	
≤235	8	5	0.099
Cholesterol (mg/dl)			
>200	5	12	
≤200	8	1	0.011
Molecular targeted therapy			
No	23	14	
Yes	11	18	0.082
Average relative dose intensity (%)			
median (range)	89.2 (50–100)	93.4 (64.3-100)	0.380

CEA: carcinoembryonic antigen; CA19-9: carbohydrate antigen 19–9.

### Prognostic factors influencing long-term survival

The correlations between progression-free survival and various clinicopathological factors are shown in Table 4. According to a univariate analysis, the progression-free survival exhibited a significant relationship with the pretreatment AGR only. In addition, a multivariate analysis indicated that only the pretreatment AGR was an independent risk factor for a poor progression-free survival. The correlations between overall survival and various clinicopathological factors are shown in Table 5. According to a univariate analysis, the overall survival exhibited a significant relationship with the pretreatment AGR and NLR. In addition, a multivariate analysis indicated that the pretreatment AGR and NLR were independent risk factors for a poor overall survival.

**Table 4:** **Correlations between progression-free survival and various clinicopathological factors**

	Univariate analysis			Multivariate analysis		
	Hazard ratio	95% CI	*p*-value	Hazard ratio	95% CI	*p*-value
Location of primary tumor (Rectum)	1.190	0.569-2.486	0.644			
Histological type (Poorly, Mucinous)	1.711	0.510-5.746	0.385	2.305	0.602-8.825	0.223
Detection of unresectable tumor (Metachronous)	1.069	0.442-2.584	0.882			
Distant metastasis except peritoneal dissemination (Yes)	1.188	0.280-5.029	0.815			
Peritoneal dissemination (Yes)	0.727	0.294-1.797	0.490	1.198	0.279-5.142	0.808
The number of organs affected by metastasis (≥2)	0.541	0.241-1.125	0.137	0.273	0.083-0.902	0.033
Pretreatment CEA (>5 ng/ml)	0.787	0.236-2.624	0.696			
Pretreatment CA19-9 (>37 U/ml)	0.862	0.403-1.845	0.702			
Molecular targeted therapy (Yes)	0.911	0.449-1.848	0.797			
Cholinesterase (<235 IU/l)	0.568	0.110-2.941	0.500			
Cholesterol (<200 mg/dl)	0.852	0.234-3.102	0.809			
NLR (>2.8)	1.342	0.619-2.911	0.457	1.090	0.434-2.737	0.855
GPS (2)	1.498	0.544-4.123	0.434	1.758	0.489-6.326	0.388
AGR (>1.25)	2.527	1.152-5.545	0.021	2.662	1.085-6.531	0.033

CI: confidence interval, CEA: carcinoembryonic antigen, CA19-9: carbohydrate antigen 19–9, NLR: neutrophil to lymphocyte ratio, GPS: Glasgow prognostic score, AGR: albumin to gobulin ratio.

**Table 5:** **Correlations between overall survival and various clinicopathological factors**

	Univariate analysis			Multivariate analysis		
	Hazard Ratio	95% CI	p-value	Hazard Ratio	95% CI	p-value
Location of primary tumor (Rectum)	0.786	0.452-1.369	0.395			
Histological type (Poorly, Mucinous)	1.251	0.533-2.940	0.607	1.735	0.667-4.513	0.259
Detection of unresectable tumor (Metachronous)	0.653	0.327-1.304	0.227			
Distant metastasis except peritoneal dissemination (Yes)	0.684	0.271-1.726	0.421			
Peritoneal dissemination (Yes)	1.411	0.771-2.582	0.264	1.888	0.641-5.561	0.249
The number of organs affected by metastasis (≥2)	1.054	0.602-1.847	0.853	0.488	0.184-1.291	0.148
Pretreatment CEA (>5 ng/ml)	1.385	0.590-3.253	0.455			
Pretreatment CA19-9 (>37 U/ml)	1.619	0.900-2.913	0.108			
Molecular targeted therapy (Yes)	0.751	0.432-1.306	0.310			
Cholinesterase (<235 IU/l)	0.915	0.289-2.890	0.879			
Cholesterol (<200 mg/dl)	1.180	0.408-3.406	0.760			
NLR (>2.8)	2.639	1.383-5.035	0.003	2.457	1.165-5.182	0.018
GPS (2)	2.558	0.900-7.269	0.078	1.237	0.466-3.287	0.670
AGR (>1.25)	1.946	1.033-3.668	0.039	2.247	1.069-4.722	0.033

CI: confidence interval, CEA: carcinoembryonic antigen, CA19-9: carbohydrate antigen 19–9, AGR: albumin to globulin ratio, NLR: neutrophil to lymphocyte ratio, GPS: Glasgow prognostic score, AGR: albumin to globulin ratio.

## Discussion

In this study, we demonstrated that the pretreatment AGR can be used as a prognostic marker for predicting the chemotherapeutic response and survival time in patients with unresectable metastatic colorectal cancer. Although several studies have shown a relationship between the AGR and the prognosis in subjects with various types of cancers [12-14], previous studies have primarily focused only on survival. Moreover, there are no clinical studies of the relationship between the AGR and the chemotherapeutic response in patients with unresectable metastatic colorectal cancer. To the best of our knowledge, this is the first study to assess the value of the AGR as a prognostic marker for predicting the chemotherapeutic response in patients with unresectable metastatic colorectal cancer who receive palliative chemotherapy.

Albumin and globulin are the two major components of serum proteins and their levels correlate with systemic inflammation [14-16]. Although the serum albumin concentration is reported to reflect the nutritional status [22], this parameter is also affected by inflammation. Under conditions of inflammation, the production of albumin by hepatocytes is suppressed due to the activation of proinflammatory cytokines, including interleukin-1, interleukin-6 and tumor necrotic factor-α [16,23,24]. Globulin includes acute-phase proteins, such as C-reactive protein, serum amyloid A, complement C3, fibrinogen and ceruloplasmin [12]. As these proteins are produced in a state of inflammation, an increased level of globulin is thought to reflect the presence of continuous systemic inflammation. Taken together, a low AGR indicates the existence of continuous systemic inflammation. It has been reported that inflammation results in increased levels of cytokines, which play an important role in tumor proliferation, progression, invasion and metastasis as well as resistance to chemotherapy [17,18,25]. Therefore, the AGR, in addition to other inflammatory markers, is considered to be a useful predictor of survival and the chemotherapeutic response in patients with various types of cancers. In this study, we also evaluated other inflammatory markers, such as NLR and GPS. These markers were also useful for predicting the overall survival. However, the progression-free survival exhibited no significant relationships with NLR/GPS. Moreover, NLR had no significant relationships with the chemotherapeutic response. The AGR was considered to be more useful than other inflammatory markers in terms of being a predictor of the chemotherapeutic outcome.

In previous studies, both the serum albumin and serum globulin concentrations have been reported to be prognostic factors for survival in patients with various types of cancers [12,21,26,27]. However, in the present study, we evaluated the status of the host based on the ratio, not levels, of these parameters for the following reasons. The concentration of the serum albumin varies readily according to changes in the volume of body fluids, such as that due to dehydration and fluid retention [14]. Using the ratio means that our results were not affected by this variability. Moreover, even in patients with a normal albumin level, the AGR has been reported to be able to identify those expected to have a poor prognosis [12]. Therefore, the AGR is considered to be a more accurate prognostic marker than the serum albumin/globulin concentrations.

In this study, we demonstrated that the AGR is associated with the disease-control and progression-free survival rates. Based on these results, we speculate that the effectiveness of chemotherapy may be decreased under conditions of inflammation; in other words, the tumor microenvironment contains many cytokines, which subsequently promote the progression of the tumor and increase resistance to chemotherapy. Patients with a low AGR are considered to be more likely to display rapid progression of the tumor. Therefore, it is recommended for such patients to receive an intensive regimen.

There are several possible limitations associated with this study. Notably, we evaluated a relatively small number of patients and the study design was retrospective. Therefore, large prospective studies should be performed to confirm our findings.

## Conclusions

The pretreatment AGR may be a useful prognostic marker in patients with unresectable metastatic colorectal cancer who receive palliative chemotherapy.

## Abbreviations

AGR: Albumin to globulin ratio
NLR: Neutrophil to lymphocyte ratio
GPS: Glasgow prognostic score
FOLFOX: 5-fluorouracil + leucovorin + oxaliplatin
CapeOX: Capecitabine + oxaliplatin
FOLFIRI: 5-fluorouracil + leucovorin + irinotecan
CEA: Carcinoembryonic antigen
CA19-9: Carbohydrate antigen 19–9
